# A systematic review on urolithiasis in small ruminants according to nutrition-dependent prevalence and outcome after surgery

**DOI:** 10.14202/vetworld.2022.809-817

**Published:** 2022-03-31

**Authors:** Marlene Sickinger, Anita Windhorst

**Affiliations:** 1 Department of Ruminants (Internal Medicine and Surgery), Faculty of Veterinary Medicine, University of Giessen, Giessen, Germany; 2 Department of Medical Informatics, Faculty of Medicine, University of Giessen, Giessen, Germany

**Keywords:** castration, long-term outcome, prevalence, small ruminants, systematic review, urolithiasis

## Abstract

**Background and Aim:**

Obstructive urolithiasis is a common disease in small ruminants with fatal outcomes if left untreated. Many methods have been established; however, long-term success rates remain unsatisfactory. Four bibliographic databases (PubMed, HeBis, Medline (OvidSP), and Web of Science) were searched to evaluate the prevalence of urolithiasis in small ruminants and long-term outcomes after surgery using a systematic review. The study aimed to give evidence-based data concerning prevalence and success rates after surgery.

**Materials and Methods:**

The analysis included 24 (total 239) peer-reviewed journal articles dealing with the prevalence of urolithiasis and 16 (total 39) concerning long-term outcomes after surgery. Literature was included if it referred to species, language, availability, and relevant statements to the specific questions, including the presence of control groups. Heterogeneity tests included *Χ*^2^, I^2^, and τ^2^, respectively. A 95% confidence interval was determined, and effects were estimated using the fixed effect model. Due to a feeding-associated bias, prevalence analysis was conducted for a sorghum-based and a corn-based feeding resulting in a weighted prevalence of 62% and 17%, respectively. Analysis of long-term outcomes after surgical interventions revealed long-term success rates of 15-77% after ultrasonographic tube cystotomy and marsupialization of the urinary bladder, respectively.

**Results:**

The prevalence of urolithiasis is strongly associated with feeding and may be calculated as 17% in corn-based rations and 62% in sorghum-based rations. Surgical interventions result in guarded to moderate long-term success rates of 15-66%. Urethral stoma and marsupialization of the bladder provide acceptable long-term success rates with 71-77% but are rather salvage techniques than accepted surgical methods, especially when used in companion animals.

**Conclusion:**

The development of urolithiasis is mainly influenced by nutrition. Effective prophylaxis of this disease should, therefore, always include advisory service for the owners. Existing surgical techniques should be critically re-evaluated concerning their long-term success rates.

## Introduction

Urolithiasis in small ruminants is a common disease in feedlots and sheep and goats that are kept as companion animals [1,2]. The etiology of this disease is thought to be multifactorial and seems to be mainly related to feeding [3]. In contrast, there is no explanation for the phenomenon, that in groups of identical feeding and environmental influences, the incidences are still not equal. Several predisposing factors, that is, castration status, breed, or species affiliation, have been proposed. However, there is a lack of significant studies [4,5]. The long-term success rates after surgery in cases of obstructive urolithiasis are somewhat limited [6]. Especially, owners of companion animals request uncomplicated and long-term solutions for restoring urinary patency.

Although well known, there is no reliable information on the general prevalence of this often fatal disease in small ruminants. Various treatment techniques are performed, but an evaluation of their effectiveness is still lacking.

This study aimed to reveal evidence-based data concerning the overall prevalence of urolithiasis in sheep and goats and the long-term success rates of several surgical techniques commonly used as therapeutic measures in obstructive urolithiasis. In general, meta-analyses or systematic reviews intend to provide scientists and practitioners with an unbiased overview of literature-based results for a particular question [7]. The main advantage of such a systematic review is combining available information concerning a specific topic. Because of the magnitude of knowledge and scientific progress, such evidence-based reviews are of extraordinary importance to achieve a state of knowledge that is up to date and within a reasonable, timely reach [7].

## Materials and Methods

### Ethical approval

The study did not deal with animals but with literature only. Therefore, there is no need for ethical approval.

### Study design, period, and location

This systematic review was performed from January to May 2018. It was conducted in the Clinic for Veterinary Medicine, Justus-Liebig-University of Giessen, Germany.

### Procedures

Systematic reviews were performed according to Cochrane’s guideline starting with formulating specific questions. The questions read as follows:

1. What is the literature-based estimated prevalence of urolithiasis in sheep and goats?
2. Which surgical technique promises the highest long-term success rates after obstructive urolithiasis?

Data were collected by searching literature within four national and international databases (HeBis, PubMed, Medline (OvidSP), and the Web of Science). Searching criteria for the above-mentioned questions were as follows:

1. Prevalence of urolithiasis (#a AND #b AND #c)
  - Incidence OR epidemiology OR occurrence OR prevalence
  - Sheep OR goat OR goats OR “small ruminant” OR “small ruminants” OR ruminant OR ruminants
  - Urolithiasis OR “urinary stone” OR “urinary stones” OR “urinary calculi”
2. Evaluation of long-term success rates (#a AND #b AND #c AND #d)
  - Surgery OR operation OR technique
  - “Success rate” OR “success rates” OR outcome
  - Sheep OR goat OR goats OR “small ruminant” OR “small ruminants” OR ruminant OR ruminants
  - Urolithiasis OR “urinary stone” OR “urinary stones” OR “urinary calculi.”

The search was performed in January 2018.

### Inclusion and exclusion criteria

**Figure-1:**
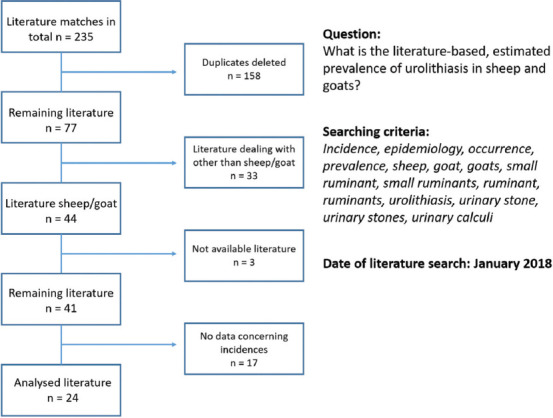
Flowchart representing the selection process through the literature review process for the prevalence of urolithiasis in small ruminants.

**Figure-2:**
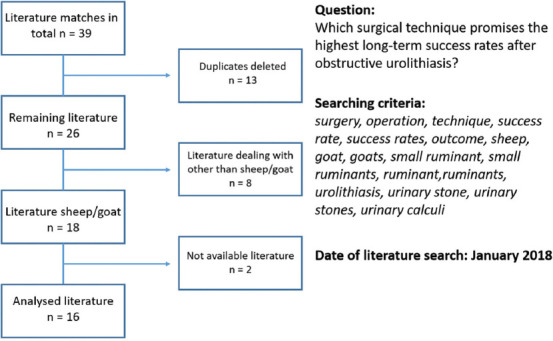
Flowchart representing the selection process through the literature review process for the long-term outcome of urolithiasis patients after surgical interventions.

After completing the search, a screening of available articles in English or German was performed. The duplicates were deleted and literature was chosen if it complied with the inclusion criteria, that are, species, language, availability, and relevant statements concerning the specific questions, including the presence of control groups. Exclusion criteria were species other than small ruminants, languages other than English or German, no disclosure concerning the questions stated above, or the lack of a control group, respectively. To select a publication, titles and abstracts were screened and full texts were read for final inclusion of the single papers. Results were merged using citation software Citavi 5.0 (Swiss Academic Software GmbH; Switzerland). Detailed overviews of deciding whether to include or exclude papers are provided in Figures-1 and 2; review protocols to register relevant data (prevalence and success rates) were generated. The analysis included 24 (total 239) peer-reviewed journal articles dealing with the prevalence of urolithiasis and 16 (total 39) concerning long-term outcomes after surgery. One of the reviewers performed a systematic evaluation of the literature.

### Assessment of bias

Depending on data availability and randomization, a bias risk assessment was performed. Apart from randomization, the bias criteria were blinding, continuity of study information, and performance of analyses. The risk of bias was defined as high if there was no randomization or if there was a loss of information during the study design due to dropouts (Tables-1 [8-11] and 2 [10,12-15]).

**Table-1:** Risk of bias assessment concerning the single studies dealing with urolithiasis prevalence in sorghum-based feeding [8-11]. Design elements leading to the risk of bias are given on the left side of the table. For example, randomization of probands led to a low risk of bias. If all animals could be evaluated at the end of the single study, there was no loss to follow-up and a low risk of bias concerning transparency of available data. The risk of the inconsistency of data is low if there is no loss to follow-up and at least 50% of the studies had random inclusion of probands.

Study no.	Design element			Risk of bias		
	
	Randomization	Blinding	Loss to follow-up	Sequence generation	Outcome assessment	Incomplete outcome data
27	Yes	Not reported	None	Low	Low	Low
28	Yes	Not reported	None	Low	Low	Low
29	Yes	Not reported	None	Low	Low	Low
32	Yes	Not reported	None	Low	Low	Low
33	Yes	Not reported	None	Low	Low	Low
36	Yes	Not reported	None	Low	Low	Low
38	Yes	Not reported	None	Low	Low	Low
39	Yes	Not reported	None	Low	Low	Low
43	Yes	Not reported	None	Low	Low	Low
51	No	Not reported	None	High	Low	Low
116	No	Not reported	None	High	Low	Low
118	No	Not reported	None	High	Low	Low
119	No	Not reported	None	High	Low	Low
123	No	Not reported	None	High	Low	Low
126	No	Not reported	None	High	Low	Low
128	No	Not reported	None	High	Low	Low
129	No	Not reported	None	High	Low	Low
133	No	Not reported	None	High	Low	Low

### Statistical analysis

Analysis of the specific review questions consisted of a 95% confidence interval (CI) assessment using the software program Bias 9.08 (bias for Windows, Epsilon publishing, Darmstadt, Germany). Homogeneity was evaluated with a *Χ*^2^-test. In case of not sufficiently homogenous studies, a configuration frequency analysis was performed to evaluate the difference of prevalence of the single studies from the overall prevalence. Concerning the success rates of different surgical techniques, statistical analysis was performed using the software program R (version 3.4.1; R Foundation for statistical computing, Vienna, Austria) and the R-package meta (version 4.9-0; R Foundation for statistical computing, Vienna, Austria). This analysis estimated the degree of heterogeneity and resulted in a calculation of estimated weight effect size.

## Results

**Figure-3:**
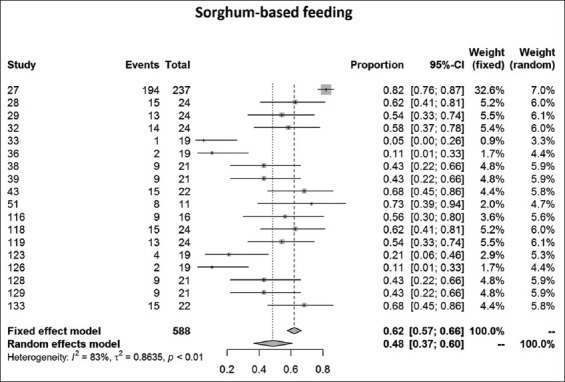
Forest plot for the combined analysis of urolithiasis prevalence in sorghum-based feeding. Heterogeneity is indexed by I^2^ and τ^2^, respectively. Percentages of urolithiasis prevalence and 95% confidence intervals are provided.

**Figure-4:**
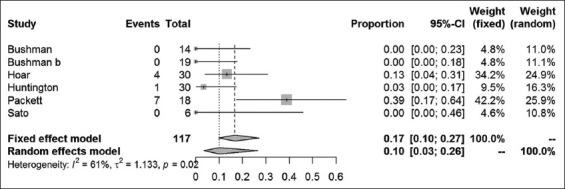
Forest plot for the combined analysis of urolithiasis prevalence in corn-based feeding. Heterogeneity is indexed by I^2^ and τ^2^, respectively. Percentages of urolithiasis prevalence and 95% confidence intervals are provided.

Because of the high variability within the study designs of feeding experiments, a direct comparison of the single studies could not be made. Therefore, in the case of two publications [8,11], the single experiments of these papers had to be evaluated separately to define homogenous experiments. These homogenous studies were then evaluated, and the effect sizes were calculated as the percentage of urolithiasis cases in relation to the number of probands within the single experiments. In doing so, two comparable feeding groups for sorghum and corn feeding could be analyzed. The risk of bias was assessed, and the 95% CIs were calculated (Tables-3 and 4) [8-15]. The analysis resulted in an estimated urolithiasis prevalence of 62% in sorghum-based rations, whereas 17% urolithiasis prevalence was displayed for corn-based feeding (Figures-3 and 4).

**Table-2:** Risk of bias assessment concerning the publications dealing with urolithiasis prevalence in corn-based feeding [10,12-15]. Design elements leading to the risk of bias are provided on the left side of the table. For example, no randomization of probands led to a high risk of bias. If all animals were evaluated at the end of the single studies, there was no loss to follow-up and a low risk of bias concerning transparency of available data was attested. The risk of the inconsistency of data seems questionable if there is no loss to follow-up, F but no randomization of probands was performed.

Study no.	Design element			Risk of bias		
	
	Randomization	Blinding	Loss to follow-up	Sequence generation	Outcome assessment	Incomplete outcome data
2	No	Not reported	None	High	Questionable	Low
18	No	Not reported	None	High	Questionable	Low
66	No	Not reported	None	High	Questionable	Low
74	Yes	Not reported	None	Low	Low	Low
117	No	Not reported	None	High	Questionable	Low
135	No	Not reported	None	High	Questionable	Low

**Table-3:** Prevalence of urolithiasis with sorghum-based feeding. The 95% confidence intervals with lower and upper limits are provided.

Author	Study no.	Uro.	Healthy	Total	Prevalence (%)	95% CI (%)	

						Lower limits	Upper limits
Crookshank *et al*. [8]	27	194	43	237	82	76.3	86.5
Crookshank *et al*. [8]	28	15	9	24	63	40.6	81.2
Crookshank *et al*. [8]	29	13	11	24	54	32.8	74.4
Crookshank *et al*. [8]	32	14	10	24	58	36.6	77.9
Crookshank *et al*. [8]	33	1	18	19	21	0	26
Crookshank *et al*. [8]	36	2	17	19	11	1.3	33.1
Crookshank *et al*. [8]	38	9	12	21	43	21.8	66
Crookshank *et al*. [8]	39	9	12	21	43	21.8	66
Crookshank *et al*. [8]	43	15	7	22	68	45.1	86.1
Davis *et al*. [9]	51	8	3	11	73	39	94
Packett and Hauschild [10]	116	9	7	16	56	29.9	80.2
Robbins *et al*. [11]	118	15	9	24	63	40.6	81.2
Robbins *et al*. [11]	119	13	11	24	54	32.8	74.4
Robbins *et al*. [11]	123	4	15	19	21	6.1	45.6
Robbins *et al*. [11]	126	2	17	19	11	1.3	33.1
Robbins *et al*. [11]	128	9	12	21	43	21.8	66
Robbins *et al*. [11]	129	9	12	21	43	21.8	66
Robbins *et al*. [11]	133	15	7	22	68	45.1	86.1

CI=Confidence interval, Uro.=Urolithiasis

**Table-4:** Prevalence of urolithiasis with corn-based feeding. The 95% confidence intervals with lower and upper limits are given.

Author	Study no.	Uro.	Healthy	Total	Prevalence (%)	95% CI (%)	

						Lower limits	Upper limits
Bushman *et al*. [12]	2	0	14	14	0	0	19.3
Bushman *et al*. [12]	18	0	19	19	0	0	14.6
Hoar *et al*. [13]	66	4	26	30	13	3,8	30.7
Huntington *et al*. [14]	74	1	29	30	3	0	17.2
Packett and Hauschild [10]	117	7	11	18	39	17,3	64.3
Sato and Omori [15]	135	0	6	6	0	0	39.3

CI=Confidence interval, Uro.=Urolithiasis

Homogeneity was tested for both feeding regimes either with the *Χ*^2^-test or the Haldane-Dawson’s U-test, because of the small number of studies that performed corn-based feeding. Both tests revealed highly significant differences within the studies (p<0.001). This heterogeneity is also displayed in Figures-3 and 4 with I^2^ of 83% and 61% for sorghum and corn, respectively.

The evaluation of which study differed from the overall average was performed using a configuration frequency analysis. After Bonferroni adjustment, differences were regarded as significant, with p<0.003 for sorghum and p<0.008 for corn feeding. Depending on the in- or exclusion of studies that differ significantly from the overall average, a combined unweighted prevalence of 48.6% and a combined weighted prevalence of 60.5% were calculated for sorghum feeding. Excluding those studies that differed significantly from the average, results in a combined unweighted prevalence of 56.1% and a combined weighted prevalence of 55.6%.

Corn-based feeding is associated with a combined unweighted prevalence of 9.2% and a weighted prevalence of 10.1%. Packett and Hauschild’s [10] study is excluded because it differed significantly with a combined unweighted prevalence of 3.2% and a weighted prevalence of 5.1%, respectively.

Graphical illustration of these results displays slightly different mean prevalence because graphics were created with the Software R (R Core Team; https://www.r-project.org), which uses different formulas to estimate the prevalence. The fixed-effects model may be chosen for homogenous studies, and weight is calculated with the inverse variance. In heterogeneous studies, the variance of all studies is corrected with τ^2^ and then calculated with the inverse variance. In summary, the estimated prevalence of urolithiasis is 48-62% in sorghum-based feeding and 10-17% in corn-based feedings.

**Figure-5:**
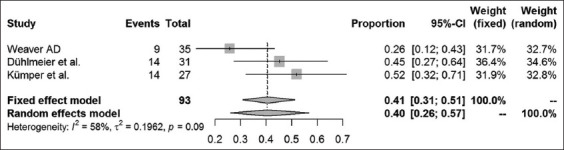
Forest plot for the combined analysis of success rates after amputation of the processus urethralis. Heterogeneity is indexed by I^2^ and τ^2^, respectively. Percentages of urolithiasis prevalence and 95% confidence intervals are provided.

**Figure-6:**
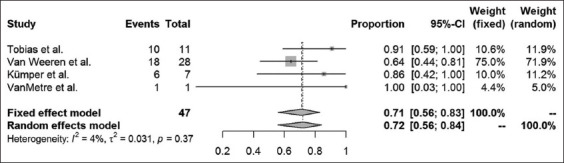
Forest plot for the combined analysis of success rates after perineal urethrostomy. Heterogeneity is indexed by I^2^ and τ^2^, respectively. Percentages of urolithiasis prevalence and 95% confidence intervals are provided.

**Figure-7:**
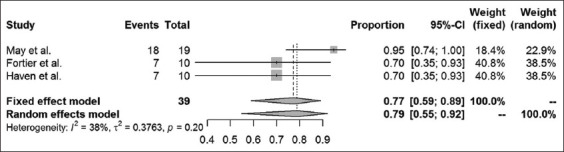
Forest plot for the combined analysis of success rates after marsupialization of the urinary bladder. Heterogeneity is indexed by I^2^ and τ^2^, respectively. Percentages of urolithiasis prevalence and 95% confidence intervals are provided.

**Figure-8:**
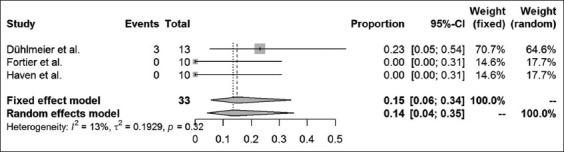
Forest plot for the combined analysis of success rates after ultrasound tube cystotomy. Heterogeneity is indexed by I^2^ and τ^2^, respectively. Percentages of urolithiasis prevalence and 95% confidence intervals are provided.

**Figure-9:**
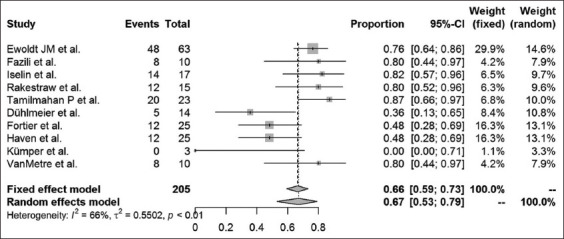
Forest plot for the combined analysis of success rates after laparotomy tube cystotomy. Heterogeneity is indexed by I^2^ and τ^2^, respectively. Percentages of urolithiasis prevalence and 95% confidence intervals are provided.

Long-term success rates were available in 16 case-control studies and case series articles. The different surgical methods used within these studies were analyzed, and results are presented for amputation of the processus urethralis, perineal urethrostomy (PU), marsupialization of the urinary bladder, ultrasound-guided tube cystotomy, and tube cystotomy through laparotomy. The results of a combined systematic review, regardless of the study type, are provided in Figures-5-9. Overall, urinary bladder marsupialization displayed the highest success rates of 77%, followed by PU with 71%, tube cystotomy through a laparotomy (66%), and the amputation of the processus urethralis with a 41% long-term success rate.

## Discussion

The performance of systematic reviews depends on the availability of high-quality, unbiased studies that deal with the same topic and is based on the same framework conditions [16]. To evaluate the prevalence of a disease, the frequency of this disease within a defined group (population) is determined for a particular time. This systematic review revealed no such literature – neither national nor international. Therefore, this examination refers to an intersection of the population consisting of animals that are housed and fed under comparable conditions, namely sorghum- and corn-based feeding rations. Still, there is a high degree of heterogeneity within the overlap. This heterogeneity is indicated by the measure I^2^ according to Higgins *et al*. [17]. As a systematic review is discouraged in cases of I^2^ being 50% or more, this study tried to evaluate a combined prevalence before and after eliminating the studies that differed significantly from the overall study mean.

Furthermore, the evaluated studies in part exhibit a non-negligible randomization bias because of group allocations depending on body weight rather than performing a randomized, blinded allocation. Although common in veterinary medicine, this method of group allocation should be discussed critically in favor of randomized clinical trials [18]. Furthermore, this systematic review deals with an epidemiologic question, which implies that classical effect size may not be determined [19], because there are no two features or treatment measures that could be compared. Therefore, the results are under reserve, especially with respect to the tests for homogeneity of higher reliability than the single studies. Thus, in the case of sorghum-based feeding, a combined prevalence for urolithiasis of 55.6% is seen. This result is more reliable than the combined prevalence for corn-based feeding (5.1%) as there was a larger number of studies providing data for sorghum-based feeding. The relevance of these results may be seen under national impact though. As sorghum and corn are common forages in the worldwide most important countries as to sheep and goat products, our results are of high international relevance. Due to lack of data, the effects of single nutrient components (especially crude protein, mineral contents, and complexing agents) on the risk of urolith development in small ruminants could not be estimated. Hydration status or daily water supply per group were also not stated and could therefore not be considered in this review. The sorghum-based feeding experiments assume an increase in urolithiasis cases, as feeding rations included cottonseed hulls and cottonseed meal. These components have high-protein concentrations and suboptimal mineral proportions that could cause urolithiasis [20]. The percentage of cottonseed components varied between 40% and 50% throughout the sorghum-based experiments, and crude protein percentages were not examined. Therefore, an analysis concerning the effect of this crude protein source could not be performed.

Besides a prophylactic optimization of nutrition, therapeutic options and surgical measures are of extraordinary importance for the owners of companion animals that are suffering from obstructive urolithiasis. As there are various surgical procedures to restore urethral patency, especially the success rates of these methods and the long-term survival rates are of high relevance for the treating veterinarian. Systematic analyses are necessary to choose the optimal therapeutic intervention. Because this systematic review did not aim to compare one surgical method to another but to show success rates of many different surgical techniques, the results are not provided as odds ratios or risk ratios [19]. This approach is deemed feasible as there is no gold standard for treating obstructive urolithiasis, although tube cystotomy in goats is considered promising [21]. However, a classical population, intervention, control, and outcomes analysis [22] comparing two interventions was not performed.

As previously discussed, the results of this analysis are subject to reservation due to the degree of study heterogeneity. Whereas studies dealing with PU, marsupialization of the urinary bladder and ultrasound-guided tube cystotomy resulted in only mild heterogeneity, the laparotomy tube cystotomy seems to be especially prone to heterogeneity (I^2^ = 66%). According to the Cochrane classification [23], 50-90% heterogeneity is considered substantial. The heterogeneity of the laparotomy studies relies on a diverse collection of patients, different surgeons performing the operations and the absence of a standard operation procedure for the single methods used. The diverse collection of patients, especially in cases of poor clinical condition on initial presentation, is related to a reduced chance of survival [6]. Nevertheless, success rates may be compared using the fixed-effect models to assign the surgical methods to their estimated combined success rates. According to the analysis, urinary bladder marsupialization represents the most effective surgical method with a combined estimated success rate of 77%. This method is followed by the PU (71%), the laparotomy tube cystotomy (66%), the amputation of the processus urethralis (41%), and the sonography-guided tube cystotomy (15%). However, this ranking neglects complications and side effects of the surgical methods mentioned above. For example, urinary bladder marsupialization always coincides with incontinence [4], which represents a severe side effect, especially to the owners of companion animals. Clarification and explanation of the consecutive custodial measures are indispensable when considering this surgical method. Due to the bias introduced by the lack of standard operating procedures for the single surgical methods, the power of the systematic review is subject to reservation. Nevertheless, this study represents a first attempt to estimate the literature-based long-term outcome rates for the above-mentioned surgical techniques.

## Conclusion

All treatment decisions have to include the scientific state of knowledge and personal, ethical, and economic interests. Acting only according to the results of meta-analyses and neglecting the side effects contradicts the true aim of evidence-based veterinary medicine [22]. It is evident that feeding influences the risk of developing urolithiasis and results in a prevalence of approximately 60% in sorghum-based and 10% in corn-based feeding regimes. Recommendable treatment measures are the PU and the laparotomy tube cystotomy. Prophylaxis mainly focuses on hydration status, a balanced diet, and optimizing housing and herd health measures [1]. Due to the data-related limitations of this systematic review, a registration of the presented results within the Cochrane’s database did not take place. Nevertheless, this report is the first attempt to evaluate the prevalence and outcomes of treatment measures in cases of urolithiasis. Systematic studies dealing with the effects of clinical aspects (i.e., duration of disease), prognostic markers, and prophylactic measures are yet to be conducted.

## Authors’ Contributions

MS and AW: Contributed to the original draft, investigation, and editing of the manuscript. MS: Collected the relevant literature and edited the manuscript. AW: Performed the statistical analysis and created the graphs. MS and AW: Revised the manuscript. Both authors have read and approved the final manuscript.

## References

[1] Scully C.M (2021). Management of urologic conditions in small ruminants. Vet. Clin. North Am. Food Anim. Pract.

[2] Sickinger M (2019). Therapeutic options of obstructive Urolithiasis in small ruminants. Tierärztl. Prax. G Grosstiere Nutztiere.

[3] Sullivan K, Freeman S, van Heugten E, Ange-van Heugten K, Wolfe B, Poore M.H (2013). Impact of two types of complete pelleted, wild ungulate feeds and two pelleted feed to hay ratios on the development of urolithogenic compounds in meat goats as a model for giraffes. J. Anim. Physiol. Anim. Nutr.

[4] Sickinger M, AlLugami A, von Pückler K, Failing K, Wehrend A (2019). Comparative ultrasonographic examination and measurements of the urethra and penis of castrated and intact male lambs. Pol. J. Vet. Sci.

[5] Chigerwe M, Heller M.C, Balcomb C.C, Angelos J.A (2016). Use of a percutaneous transabdominal catheter for management of obstructive urolithiasis in goats, sheep, and potbellied pigs:69 cases (2000-2014). J. Am. Vet. Med. Assoc.

[6] Riedi A.K, Nathues C, Knubben-Schweizer G, Nuss K, Meylan M (2018). Variables of initial examination and clinical management associated with survival in small ruminants with obstructive urolithiasis. J. Vet. Intern. Med.

[7] Giuffrida M.A (2017). Practical application of evidence-based practice. Vet. Clin. North Am. Exot. Anim. Pract.

[8] Crookshank H.R, Robbins J.D, Kunkel H.O (1967). Relationship of dietary mineral intake to serum mineral level and the incidence of urinary calculi in lambs. J. Anim. Sci.

[9] Davis W.D, Scott J.R, Crookshank H.R, Spjut H.J (1969). Factors affecting experimentally induced calculous disease in sheep. J. Urol.

[10] Packett L.V, Hauschild J.P (1964). Phosphorus, calcium and magnesium relationships in ovine urolithiasis. J. Nutr.

[11] Robbins J.D, Kunkel H.O, Crookshank H.R (1965). Relationship of dietary mineral intake to urinary mineral excretion and incidence of urinary calculi in lambs. J. Anim. Sci.

[12] Bushman D.H, Emerick R.J, Embry L.B (1964). Incidence of urinary calculi in sheep as affected by various dietary phosphates. J. Anim. Sci.

[13] Hoar D.W, Emerick R.J, Embry L.B (1969). Ovine phosphatic urolithiasis as related to the phosphorus and calcium contents and acid-base-forming effects of all-concentrate diets. J. Anim. Sci.

[14] Huntington G.B, Emerick R.J, Embry L.B (1977). Sodium bentonite or sodium bicarbonate as aids in feeding high-concentrate diets to lambs. J. Anim. Sci.

[15] Sato H, Omori S (1977). Incidence of urinary calculi in goats fed a high phosphorus diet. Japanese J. Vet. Sci.

[16] Sargeant J.M, O'Connor A.M (2020). Scoping reviews, systematic reviews, and meta-analysis:Applications in veterinary medicine. Front. Vet. Sci.

[17] Higgins J.P.T, Thompson S.G, Deeks J.J, Altman D.G (2003). Measuring inconsistency in meta-analyses. Biometric. J.

[18] Wareham K.J, Hyde R.M, Grindlay D, Brennan M.L, Dean R.S (2017). Sponsorship bias and quality of randomised controlled trials in veterinary medicine. BMC Vet. Res.

[19] O'Connor A.M, Sargeant J.M, Wang C (2014). Conducting systematic reviews of intervention questions III:Synthesizing data from intervention studies using systematic review. Zoonoses Public Health.

[20] Sun W.D, Wang J.Y, Zhang K.C, Wang X.L (2010). Study on precipitation of struvite and struvite-K crystal in goats during the onset of urolithiasis. Res. Vet. Sci.

[21] Videla R, van Amstel S (2016). Urolithiasis. Vet. Clin. North Am. Food Anim. Pract.

[22] Schmidt P.L (2007). Evidence-based veterinary medicine:Evolution, revolution, or repackaging of veterinary practice?. Vet.Clin. North Am. Small Anim. Pract.

[23] https://handbook-5-1.cochrane.org.

